# Supplementary material for: Unraveling the functional role of DNA demethylation at specific promoters by targeted steric blockage of DNA methyltransferase with CRISPR/dCas9
Source: Nat Commun. 2021 Sep 29;12:5711. doi: 10.1038/s41467-021-25991-9 (PMC8481236; doi:10.1038/s41467-021-25991-9)
Supplement: Supplementary file 1 — Supplementary Information [file 41467_2021_25991_MOESM1_ESM.pdf]

Supplementary Information for: Unraveling the functional role of DNA demethylation at specific promoters by targeted steric blockage of DNA methyltransferase with CRISPR/dCas9

Sapozhnikov & Szyf

This supplementary information contains Supplementary Figures 1-11 and Supplementary Tables 1-7.

Supplementary Figure 1

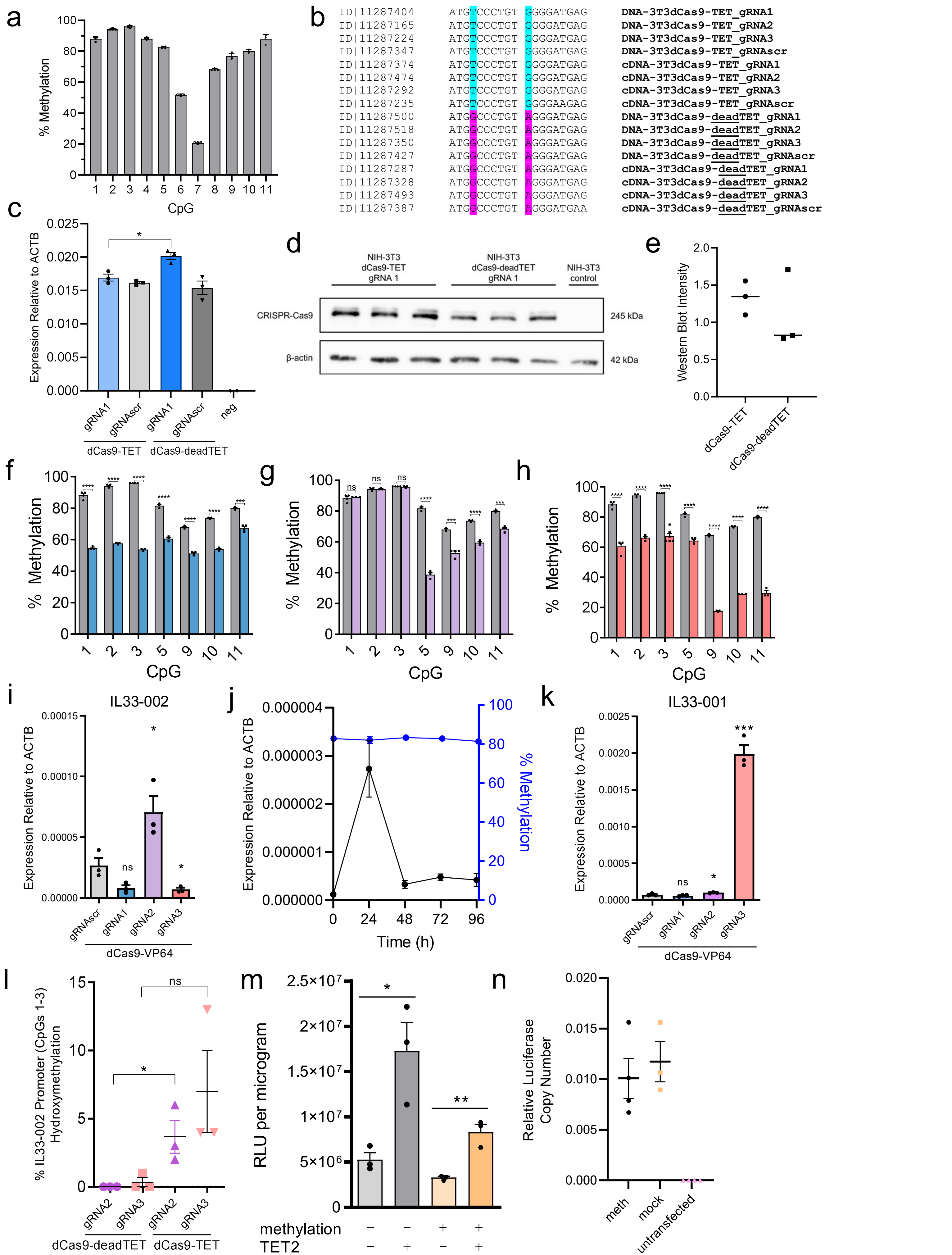

Supplementary Figure 1. Confounds involved in CRISPR/TET-based approaches. (A) Percent of DNA methylation (mean ± SEM) assayed by bisulfite-pyrosequencing at 11 CpGs in the *Il33-002* promoter of control, untreated NIH-3T3 cells (*n* = 3 independent samples for CpGs 1-8, *n* = 4 independent samples for CpGs 9-11). (B) Aligned Sanger sequencing results of region of TET bearing the inactivating mutation in deadTET controls from one representative cell line from triplicate treatments in Fig. 1 with Sanger ID (left), DNA sequence (middle), and source of DNA (right). (C) mRNA expression (mean ± SEM) of dCas9-TET or dCas9-deadTET (single primer pair that amplifies common region) relative to *Actb* in corresponding NIH-3T3 cells from Fig.1E-I expressing either dCas9-TET or dCas9-deadTET in combination with either gRNAscr or gRNA1. Negative control cells are untreated NIH-3T3 (*n* = 3 independent samples, *n* = 2 independent samples for negative control). (D) Western blot with anti-CRISPPR/Cas9 (top panel) or anti-β-actin (bottom panel) antibody in NIH-3T3 cells expressing dCas9-TET and gRNA1 (*n* = 3 independent experiments), dCas9-deadTET and gRNA1 (*n* = 3 independent experiments), or negative control (untreated) NIH-3T3 cells (*n* = 1). (E) Quantification of (D) using ImageJ involving normalization of anti-CRISPR/Cas9 antibody signals to anti-β-actin antibody signals (mean, *n* = 3 independent experiments) (F-H) Percent of DNA methylation assayed by bisulfite-pyrosequencing at 7 targeted CpGs in NIH-3T3 cells treated with dCas9-VP64 and either gRNA1 (F; blue, *n* = 3-5 independent experiments), gRNA2 (G; purple, *n* = 3-4 independent experiments), gRNA3 H; pink, *n* = 3-6 independent experiments) or gRNAscr (grey; identical data in B-D, shown for comparison) (mean ± SEM) (*n* varies depending on specific condition and CpG; see Source Data file for specific *n* of interest). (I) Expression of *Il33-002* (mean ± SEM) quantified by RT-qPCR and normalized to *Actb* expression in NIH-3T3 stably expressing one of 4 gRNAs and dCas9-VP64. Statistical comparisons are to gRNAscr condition (*n* = 3 independent experiments). (J) (Left y-axis) Il33-002 gene expression (mean ± SEM) quantified by RT-qPCR and normalized to *Actb* expression in NIH-3T3 cells stably expressing dCas9-VP64 and transiently transfected with gRNA2 and harvested after the indicated number of hours. (Right y-axis) DNA methylation level (mean ± SEM) of CpG 11 at the TSS of the *Il33-002* promoter in the corresponding cells (*n* = 3 independent samples). (K) Expression of *Il33-001* (mean ± SEM) quantified by RT-qPCR and normalized to *Actb* expression in NIH-3T3 stably expressing one of 4 gRNAs and dCas9-VP64. Statistical comparisons are to gRNAscr condition (*n* = 3 independent experiments). (L) Percent of DNA hydroxymethylation (mean ± SEM) assayed by $KRuO_4$ oxidation of DNA followed by bisulfite-pyrosequencing in parallel with unoxidized controls and calculated as decrease in methylation after oxidation at CpGs 1, 2, and 3, averaged, which are distant from the gRNA2 (purple) and gRNA3 (pink) binding sites, under the specified stable treatments in NIH-3T3 cells (x-axis) (*n* = 3 independent experiments). (M) Relative light units normalized to extracted total protein quantity (mean ± SEM) in transfected HEK293 cells. Cells were transiently transfected with methylated or unmethylated SV40-luciferase vector along with mammalian TET2 expression plasmid or empty vector (pcDNA3.1) control (*n* = 3 independent experiments) (N) Cellular luciferase gene (DNA) copy number (mean ± SEM), measured by qPCR with primers in Supplementary Table S2, and normalized to levels of genomic *Actb*, for untransfected cells and transfections of 50 ng of SV40-pCpGI, either mock methylated or fully methylated by M.SssI (*n* = 3 independent experiments). * indicates statistically significant difference of $P < 0.05$, ** of $P < 0.01$, *** of $P < 0.001$, **** of $P < 0.0001$, and ns = not significant (Student's t-test, two-sided, with Holm-Sidak correction if number of tests is greater than 3). Source data are provided as a Source Data file.

Supplementary Figure 2

a

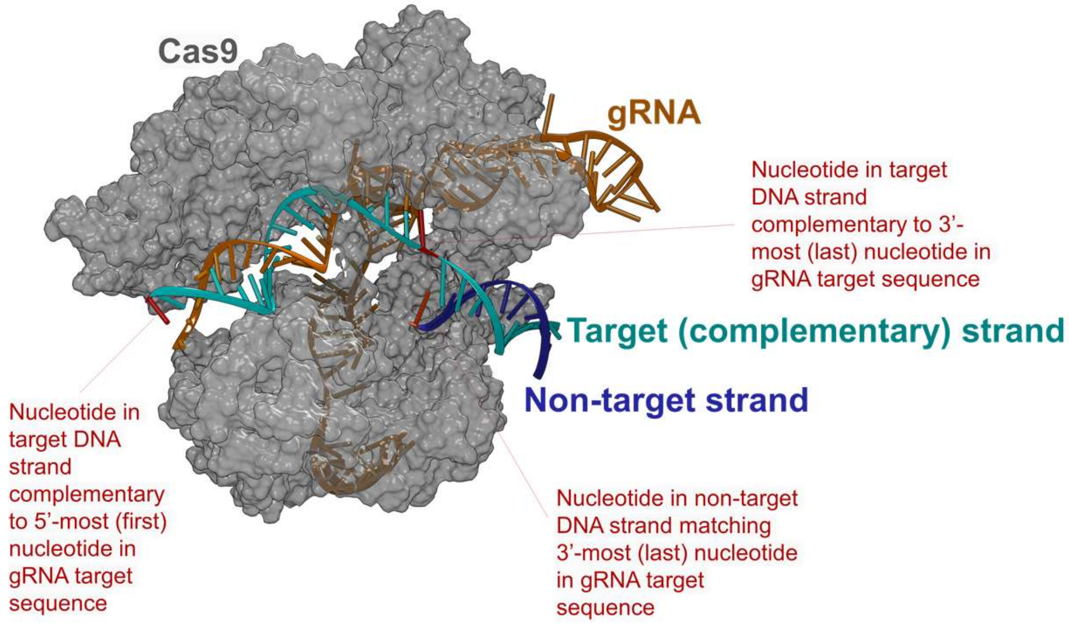

b

```
                                 3'-RRGNNACGACGAGGGGCGGCGGGCGA-5'              SAgRNA4(SPgRNA4)
                      3'-RRGNNGGCGGCCGAGGTACGACGAGG-5'                         SAgRNA3(SPgRNA3)
                     3'-RRGNNCGGCGGCCGAGGTACGACGAG-5'                          SAgRNA2(SPgRNA2)
                    3'-RRGNNGCGGCGGCCGAGGTACGACGA-5'                           SAgRNA1(SPgRNA1)
CGAAGGCTCCATGCTGCTCCCCGCCGCCGGCTCCATGCTGCTCCCCGCCGCCCGCTGCCTGCTCTCCCCCTCTCCGC
19                    20 21 22                     23 24  25                         26      CpG#
17                    16 15 14                     13 12  11                         10      CpG# in Fig. 3
```

c

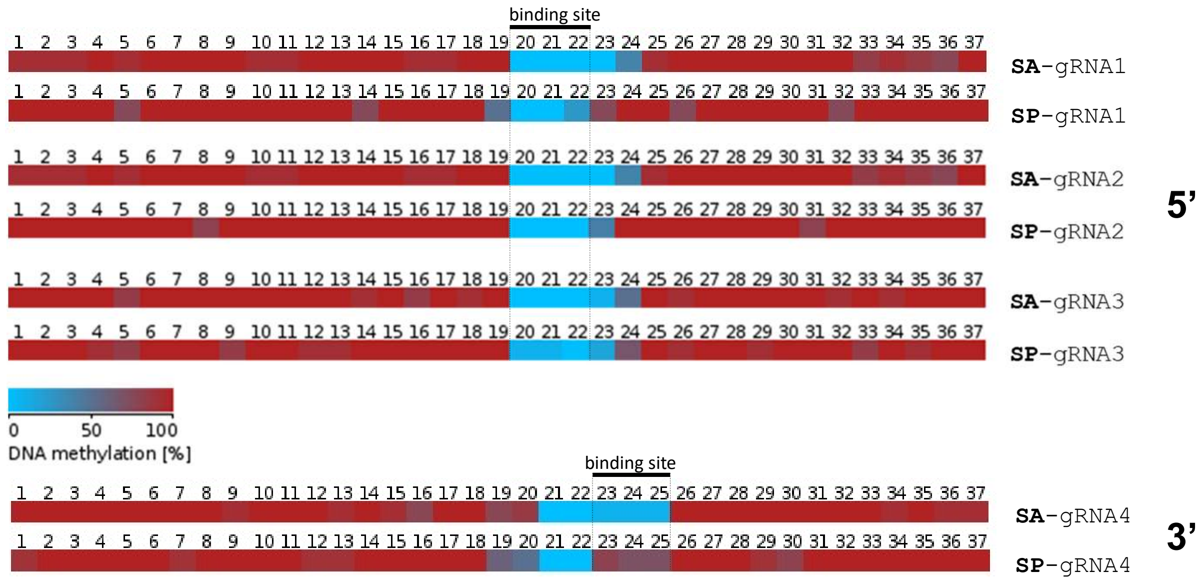

d

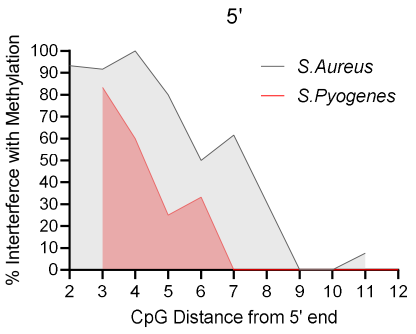

e

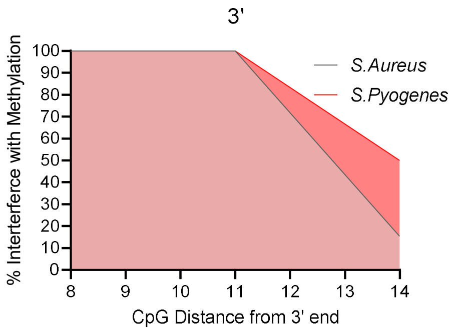

Supplementary Figure 2. Differences in steric interference with DNA methyltansferases by Cas orthologs. (A) Cryo-EM structure of Cas9 (grey) in complex with gRNA (orange), target DNA strand (light blue), and non-target DNA strands (dark blue) (partial) from [42] (PDB: 6O0Z). Two 5' prime nucleotides (CC in target DNA strand, GG in gRNA) that were not resolved in the cryo-EM structure were built and energy minimized using Molecular Operating Environment (MOE) software. The final figure was generated in UCSF Chimera. Red nucleotides are labeled accordingly and represent the nucleotides in the DNA complementary to either the first (5') nucleotide of the gRNA target sequence or the last (3') nucleotide of the gRNA target sequence as well as its complement in the non-target strand. (B) Diagram and sequence of *CDKN2A* region targeted by additional gRNAs. Due to the display of the reverse complement sequence of Fig. 3, CpGs are numbered differently (black) but Fig. 3 numbering system is shown below in red. gRNA sequences are shown in green, where the entire green sequence represents the *S. pyogenes* gRNA and the addition 5' nucleotide in black represents the additional nucleotide needed for *S. aureus* gRNAs. *S. aureus* PAM site is shown in blue, with the first 3 nucleotides (5' to 3') represent the NGG PAM of the *S. pyogenes* gRNA. (C) Each horizontal row depicts a heatmap of average DNA methylation at each numbered CpG over 10-20 (except SP-gRNA4, 4 clones) individual strands of DNA (bisulfite-converted clones) where light blue represents 0% methylation and dark red represents 100% methylation. The CpGs within the binding site of the labeled gRNA are labeled and enclosed in dashed lines. gRNAs1-3 interrogate DNA methylation interference of 5' proximal CpGs and gRNA4 interrogates that of 3' proximal CpGs. Lowly methylated strands of DNA (poor M.SssI methylation) and strands with unaffected binding sites (unbound by dCas9) were excluded from the analysis because efficacy was not under evaluation. (D-E) Data from (C) transformed into a percent methylation as a function of CpG distance in base pairs from the 5' (D) or 3' (E) end of the gRNA sequence (including PAM) and *S. aureus*(grey) or *S. pyogenes* (pink) across gRNAs 1-3 (D) or gRNA4 (E). Source data are provided as a Source Data file.

Supplementary Figure 3

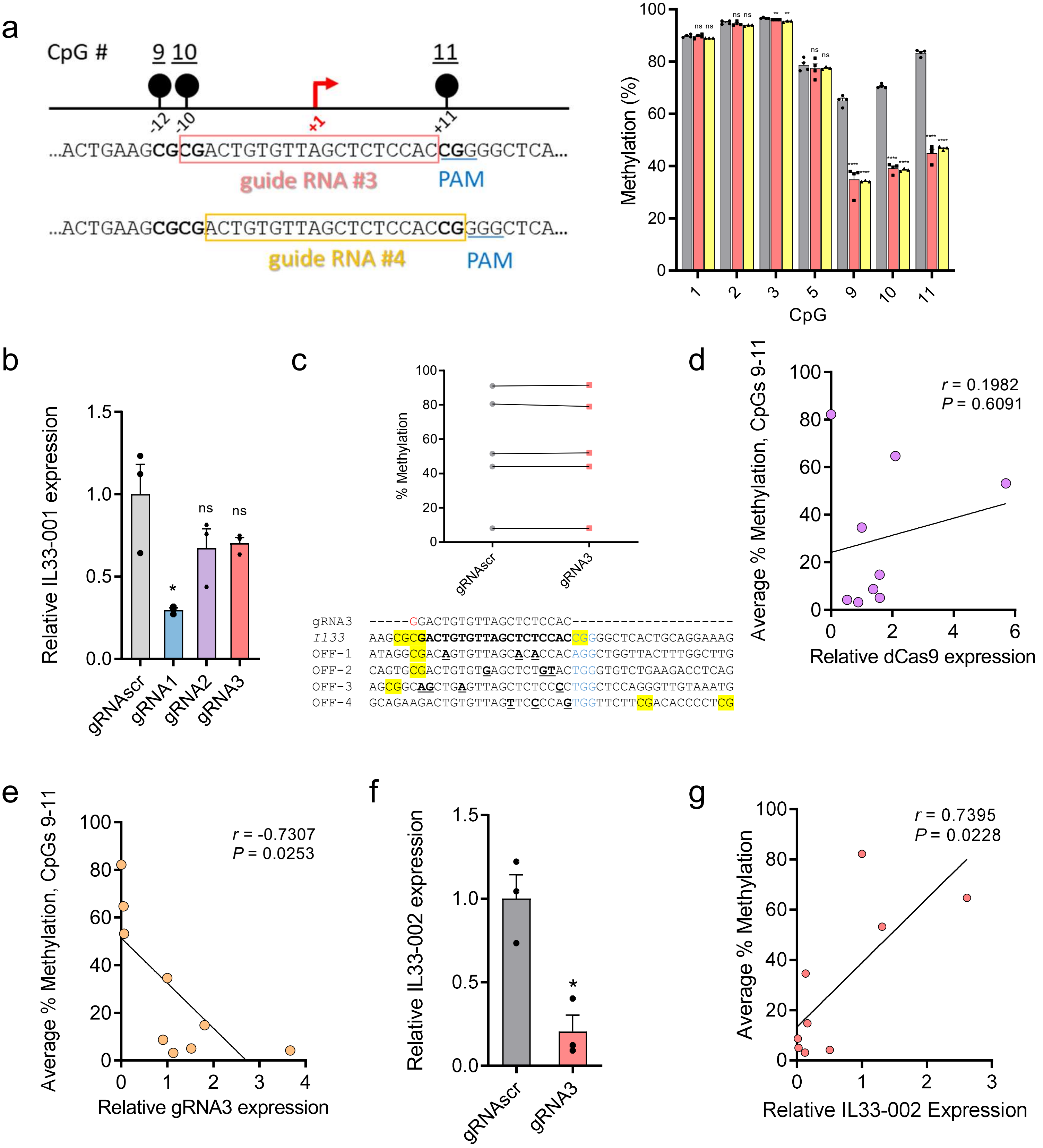

Supplementary Figure 3. Characteristics of dCas9-based inhibition of methylation at the *Il33* locus. (A) (Left) Diagram of *Il33-002* promoter with location of gRNA3 and gRNA4. TSS is marked by a red arrow and CpGs are marked by black circles. In sequence below, PAM represents protospacer adjacent motif, gRNA sequences are boxed, and CpGs are bolded. (Right) Methylation levels (mean ± SEM) assessed by pyrosequencing of NIH-3T3 cells expressing dCas9 and gRNA3 (pink) or gRNA4 (yellow). Values displayed as mean +/- SEM (n = 3 independent experiments). (B) *Il33-001* expression (mean ± SEM) in NIH-3T3 cell lines stably expressing gRNAscr or one of 3 *Il33-002*-targeting gRNAs in combination with dCas9, assayed by qRT-PCR and normalized to *Actb* expression (n = 3 biologically independent samples). (C) Comparison of methylation levels, assayed by pyrosequencing, of 5 top off-target CpGs in NIH-3T3 cell lines stably expressing scrambled gRNA or gRNA 3 in combination with dCas9. (D) Correlation of 9 dCas9:gRNA3 clones from (Fig 4E); x-axis displays dCas9 expression normalized to *Actb* expression; y-axis displays average methylation at CpGs 9, 10, and 11 in each clone assayed by pyrosequencing (r = 0.1982, P = 0.6091). (E) Correlation of 9 dCas9:gRNA3 clones from (Fig 4E); x-axis displays gRNA3 expression normalized to *Actb* expression; y-axis displays average methylation at CpGs 9, 10, and 11 in each clone assayed by pyrosequencing (r = -0.7307, $P < 0.05$). (F) *Il33-002* expression (mean ± SEM) in NIH-3T3 cell lines stably expressing scrambled gRNA or gRNA3 in combination with dCas9, assayed by qRT-PCR and normalized to *Actb* expression (n = 3 biologically independent experiments, * indicates $P < 0.05$ vs gRNAscr, t-test). (G) Scatter plot of 9 dCas9:gRNA3 clones from (4E); x-axis displays relative *Il33-002* expression as assayed in (F); y-axis displays average methylation at CpGs 9, 10, and 11 in each clone assayed by pyrosequencing (r = 0.7395, P = 0.0228). * indicates statistically significant difference of $P < 0.05$, ** of $P < 0.01$, *** of $P < 0.001$, **** of $P < 0.0001$, and ns = not significant (Student's t-test, two-sided, with Holm-Sidak correction if number of tests is greater than 3). Source data are provided as a Source Data file.

Supplementary Figure 4

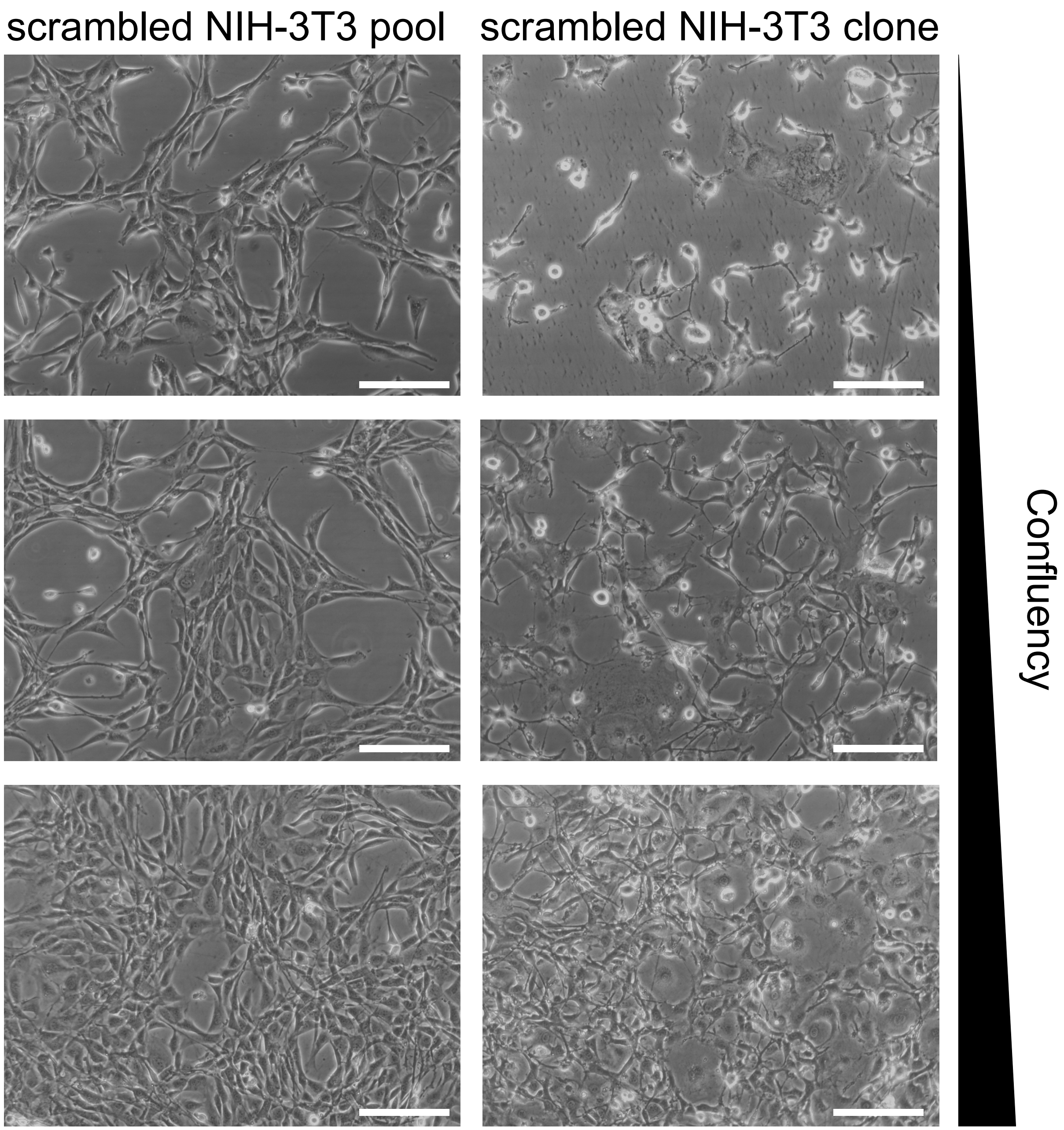

Supplementary Figure 4. Clonal selection is a deficient method for derivation of cell lines with effective dCas9-based demethylation. Light microscopy images demonstrating the appearance of normal healthy NIH-3T3 pools (3 left panels) with increasing confluency downwards. In comparison, morphological irregularities can be seen after clonal isolation from the same source cells in 3 distinct clonal populations at 3 different levels of confluency (*n* = 1 independent clonal cell lines for each level of confluency for a total of 3 independent cell lines per experimental condition). Scale bars represent 100 μM.

Supplementary Figure 5

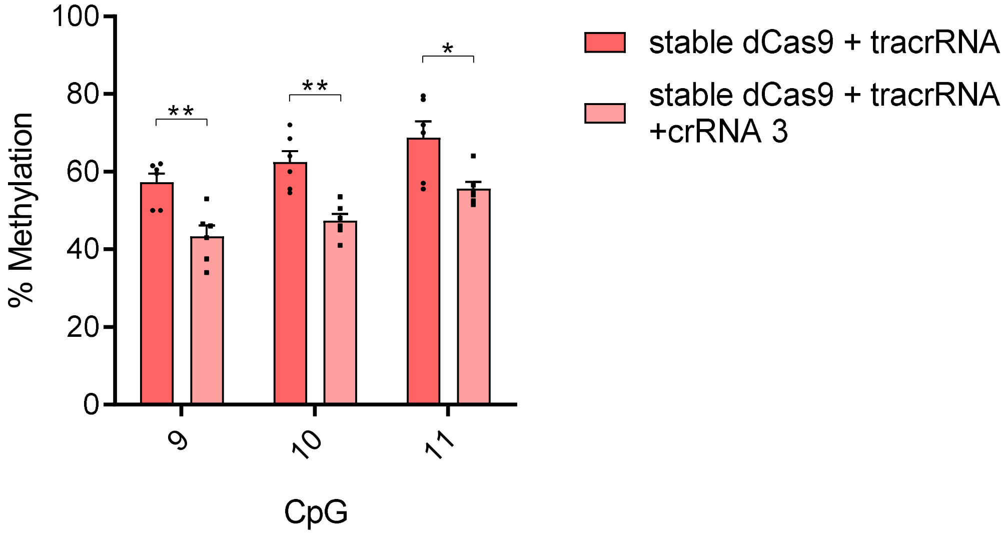

Supplementary Figure 5. Transient transfection of gRNA components. Methylation levels assessed by bisulfite-pyrosequencing (mean ± SEM) of target CpGs 9, 10, and 11 after NIH-3T3 cells stably expressing dCas9 were transiently transfected (Xtremegene siRNA transfection reagent, Sigma) with either tracrRNA alone (red) or tracrRNA and crRNA3 (pink), a two-component version of gRNA3 (n = 6 biologically independent experiments). * indicates statistically significant difference of $P < 0.05$ and ** of $P < 0.01$(Student's t-test, two-sided, with Holm-Sidak correction). Source data are provided as a Source Data file.

Supplementary Figure 6

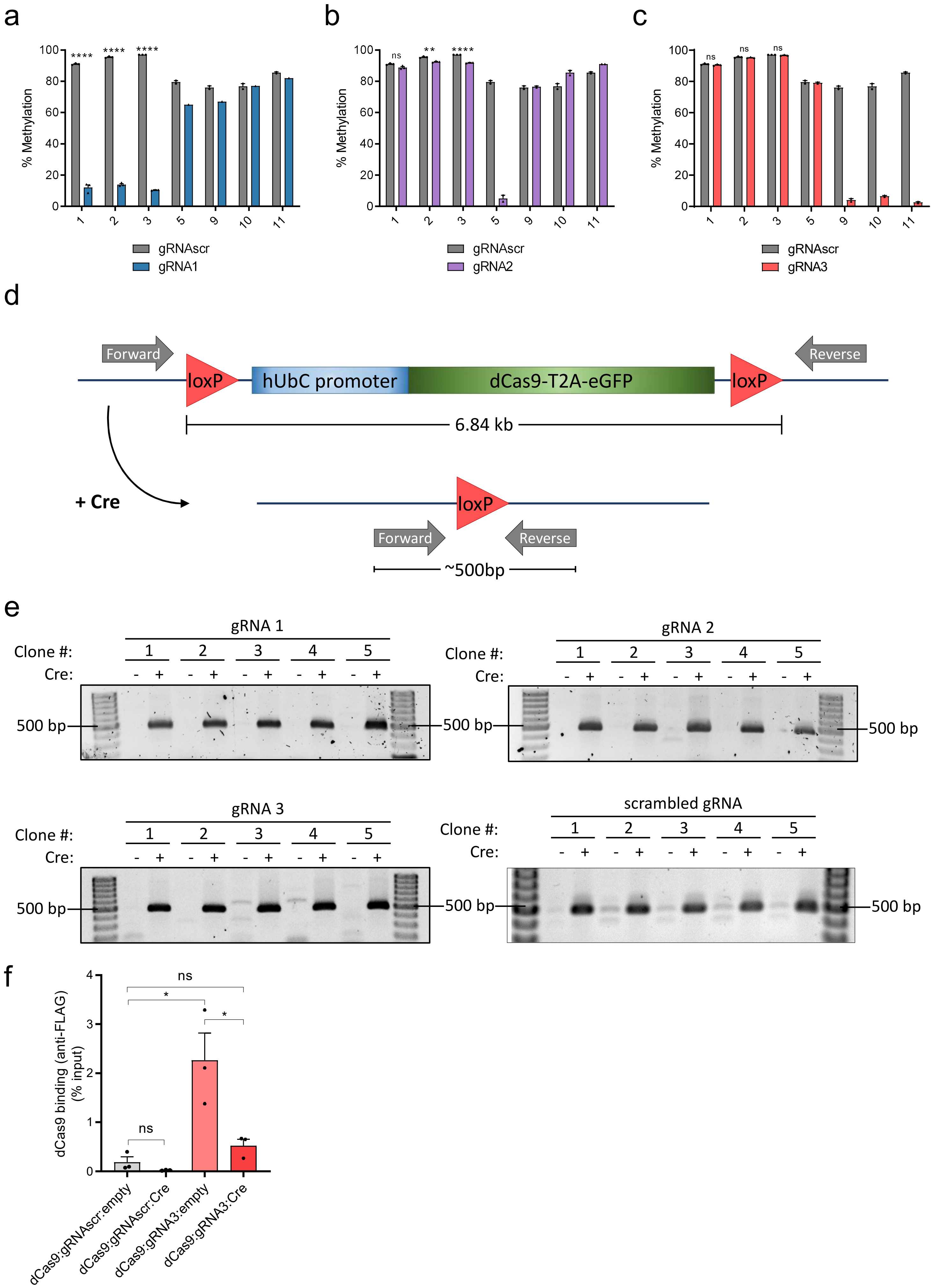

Supplementary Figure 6. Verification of success of removable lentiviral dCas9 strategy. (A-C) DNA methylation levels (mean ± SEM) by pyrosequencing of NIH-3T3 cells stably expressing gRNA1 (A), gRNA2 (B), or gRNA3 (C) and floxed dCas9 in order to validate targeted demethylation (*n* = 1-3 biologically independent samples). (D) Diagram of the dCas9 expression construct. It is flanked by loxP sites that facilitate recombination and deletion by Cre recombinase. Forward and reverse PCR primers lie outside the loxP sites such that a 6.84 kb product could be made when Cre is not present in the cells and if PCR extension times are increased to allow this product to form. After removal of the dCas9 expression cassette by Cre recombinase, the same PCR primers create a product of approximately 500 base pairs in size. (E) Agarose gels showing recombination-dependent PCR products using the primers in (D) in *n* = 5 independent cell lines stably expressing dCas9 and each indicated gRNA after independent treatment by empty virus (-) or Cre recombinase (+). A 500 base pair product is visible in each Cre-containing lane. Primers are listed in Supplementary Table S2. (F) Chromatin immunoprecipitation of cells from Fig. S6E with antibody against the 5' 3XFlag-tagged dCas9 (anti-Flag antibody) followed by qPCR using primers surrounding the *Il33-002* TSS. dCas9 binding is expressed as percent input (*n* = 3 biologically independent experiments). * indicates statistically significant difference of $P < 0.05$, ** of $P < 0.01$, *** of $P < 0.001$, **** of $P < 0.0001$, and ns = not significant (Student's t-test, two-sided, with Holm-Sidak correction if number of tests is greater than 3). Source data are provided as a Source Data file.

Supplementary Figure 7

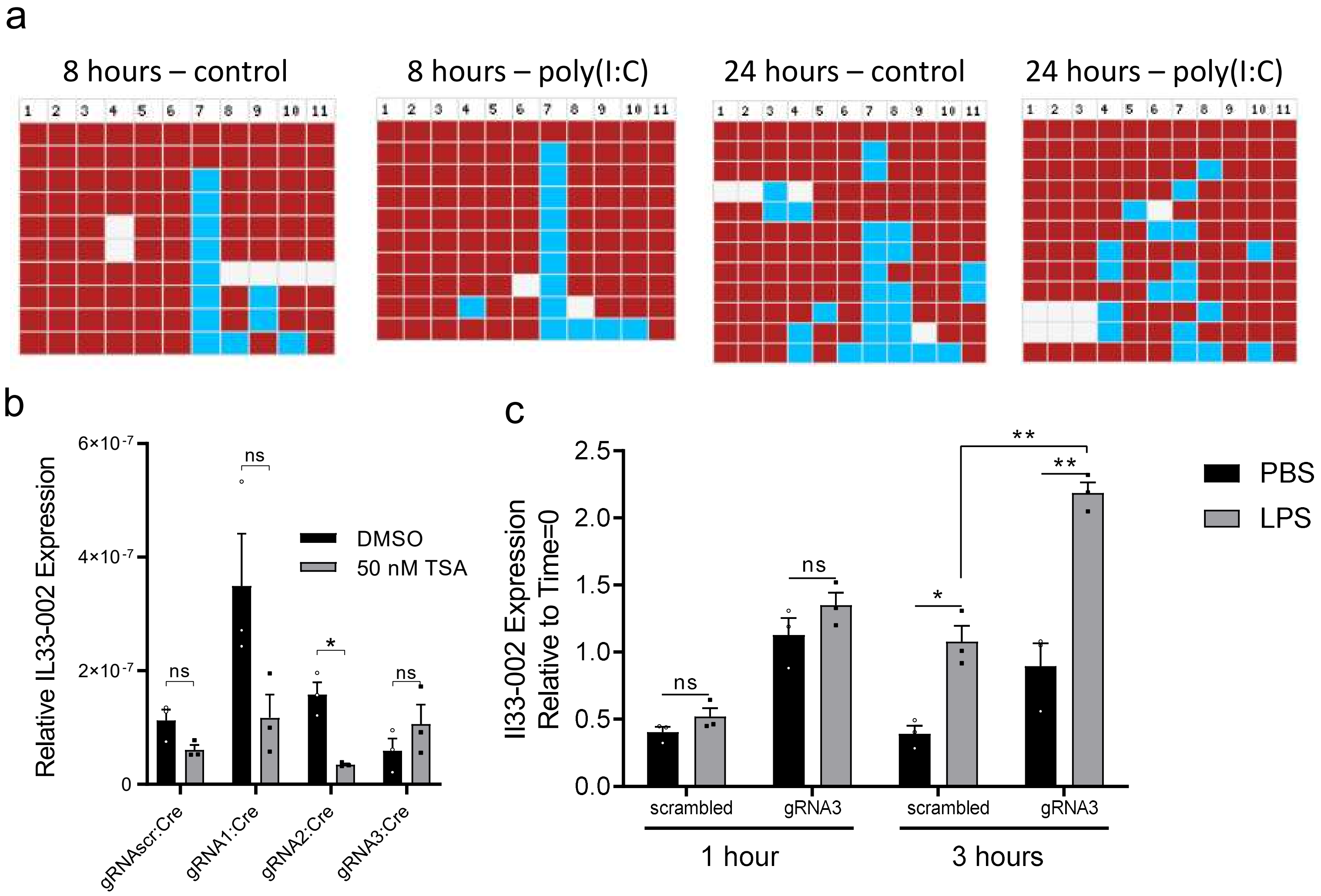

Supplementary Figure 7. Effect of inducing agents on *Il33*. (A) Bisulfite-cloning and sanger sequencing analysis of the *Il33-002* promoter in NIH-3T3 cells treated with 1 µg/mL poly(I:C) or water control for 8 or 24 hours. Each horizontal row is one strand of DNA. Numbers indicate the CpG in the promoter. Red squares indicate methylated CpGs, blue squares indicate unmethylated CpGs, and white squares indicate a lack of data due to sequencing failure. (B) *Il33-002* expression (mean ± SEM) in 50nM TSA or vehicle (DMSO) treated NIH-3T3 cell lines stably expressing gRNAscr, gRNA1, gRNA2, or gRNA3 under high-puromycin conditions in combination with dCas9, followed by dCas9 removal by Cre recombinase as assayed by qRT-PCR and normalized to *Actb* expression (*n* = 3 biologically independent experiments). (C) *Il33-002* expression (mean ± SEM) in 100 ng/mL lipopolysaccharide (LPS) or vehicle (PBS) treated NIH-3T3 cell lines stably expressing gRNAscr or gRNA3 under high-puromycin conditions in combination with dCas9, followed by dCas9 removal by Cre recombinase, as assayed by qRT-PCR and normalized to *Actb* expression, either 1 or 3 hours after treatment and displayed relative to expression measured at time=0 (*n* = 3 biologically independent experiments). * indicates statistically significant difference of $P < 0.05$, ** of $P < 0.01$, *** of $P < 0.001$, **** of $P < 0.0001$, and ns = not significant (Student's t-test, two-sided, with Holm-Sidak correction if number of tests is greater than 3). Source data are provided as a Source Data file.

Supplementary Figure 8

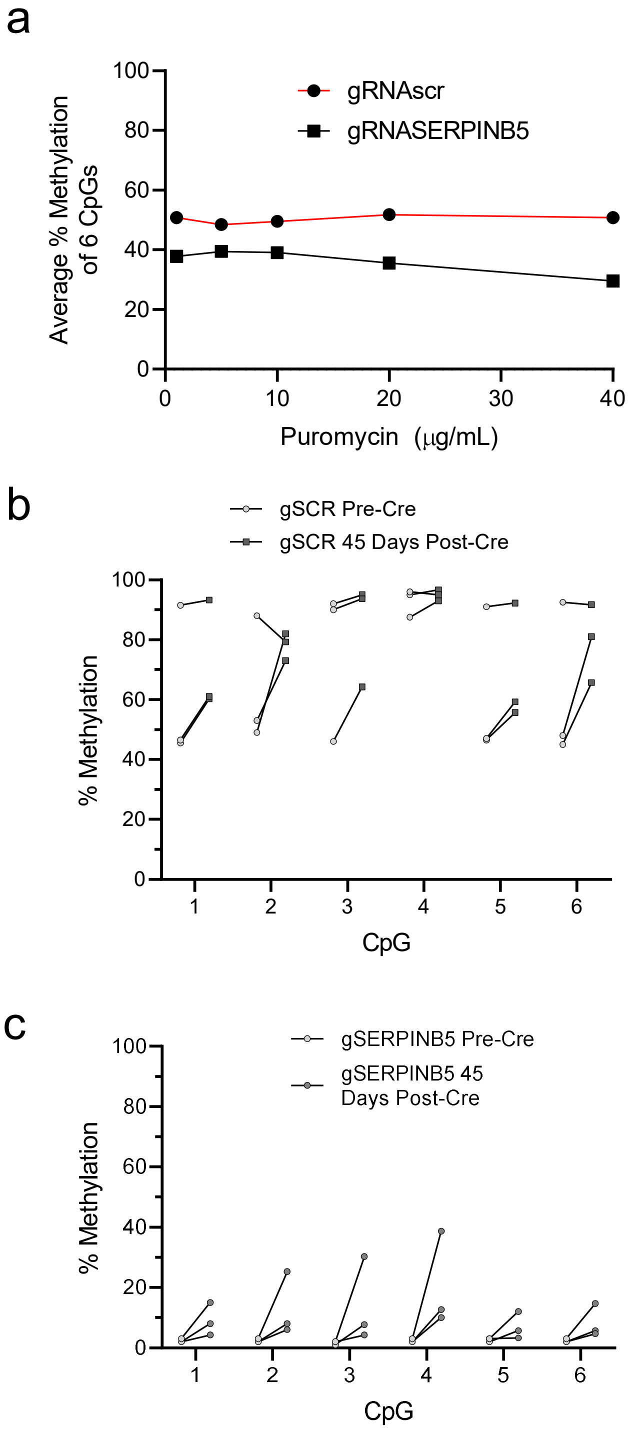

Supplementary Figure 8. Methylation levels of *SERPINB5*. (A) DNA methylation levels (mean ± SEM) assessed by bisulfite-pyrosequencing of CpGs 1-6 in the *SERPINB5* promoter in MDA-MB-231 cell lines stably expressing dCas9 and either gRNAscr (red) or gRNASERPINB5 (black), averaged across all 6 CpGs and plotted as a function of increasing puromycin concentration (*n* = 1 per puromycin concentration). (B-C) Percent DNA methylation assessed by bisulfite-pyrosequencing of *n* = 3 MDA-MB-231 clonal cell lines expressing dCas9 and gSCR (B) or gRNASERPINB5 (C) prior to dCas9 excision by Cre (light grey) and 45 days after the end of selection for Cre recombinase (dark grey) (mean ± SEM). Source data are provided as a Source Data file.

Supplementary Figure 9

a

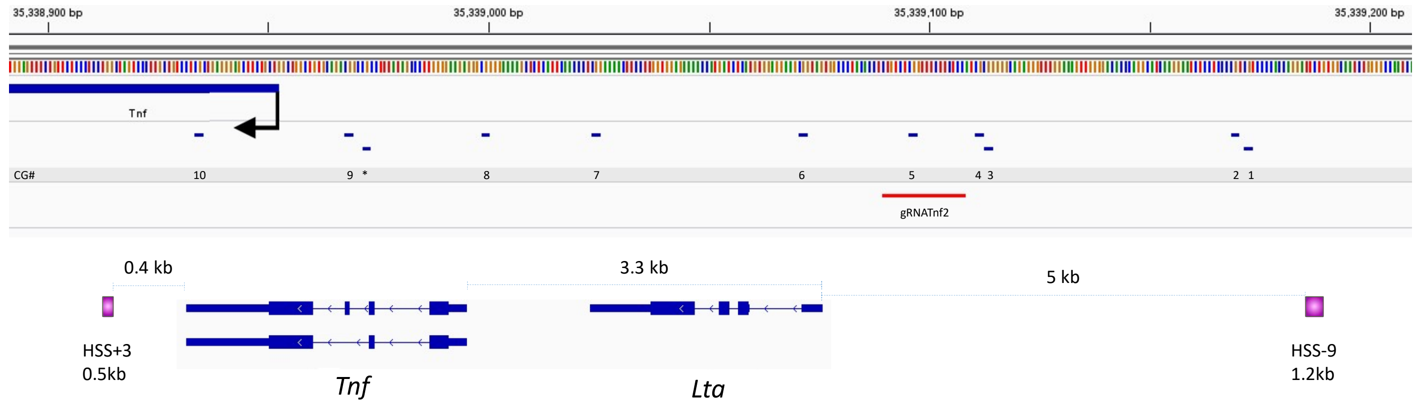

b

gRNA	gRNAscr	1	2	3	4	5	6
CpG target		1,2	3 to 5	6	7	8,10	11
CpG							
1	49	3.33	2.7	1.7	43.7	51	30
2	58	4.33	3	2.3	48.3	60	28.3
3	93.7	58	4.3	93.7	79.7	93	90.3
4	88.3	81	6	86.7	72.7	88.3	81.3
5	96.7	65.3	4	92	87.3	88.7	94.3
6	93.7	64.7	7.7	28	93.6	81.7	83
7	38.7	31.7	2	37.7	23.7	51.7	40.7
8	58.3	92	6	11	52.3	52	52.3
9	90.3	83.7	5	22	85.6	77.3	91.3
10	84.7	68	6.7	71.3	60	83.7	66

c

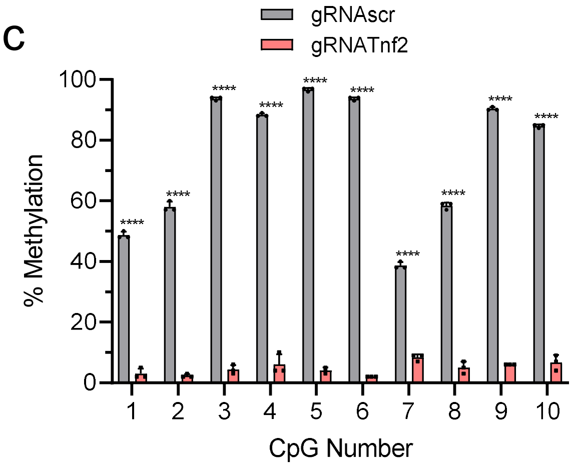

d

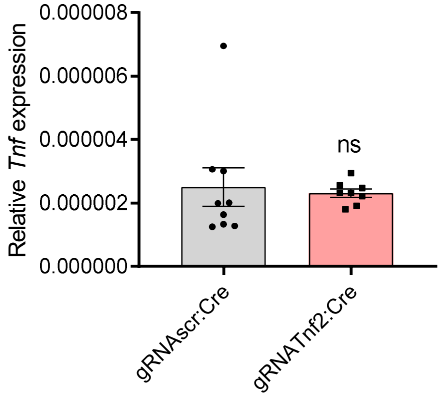

Supplementary Figure 9. Demethylation of the Tnf promoter. (A) Genome browser view of the murine Tnf locus;(Top) each CG location marked by a blue dash and numbered below, TSS indicated by a black arrow., and the location of gRNATnf2 is labeled with a red line and marked accordingly; (Bottom) Two known distal enhancers of Tnf expression indicated with purple boxes, named and marked with distances to Tnf TSS [77]. (B) Table demonstrating the average methylation of Tnf CpGs numbered in (A) as measured by bisulfite-pyrosequencing a function of six candidate Tnf-targeting gRNAs or gRNAscr control in NIH-3T3 cells also stably expressing dCas9. CpGs within the gRNA binding site are indicated below the gRNA number and their methylation status is highlighted in yellow in the corresponding gRNAs. (C) DNA methylation levels (mean ± SEM) assessed by bisulfite-pyrosequencing of NIH-3T3 cells stably expressing dCas9 and either gRNATnf2 (pink) or gRNAscr (grey) (n = 3 biologically independent experiments). (D) Tnf expression (mean ± SEM) in NIH-3T3 cell lines subcloned from those in Fig. 7H stably expressing gRNAscr (grey) or gRNATnf2 (pink) under high-puromycin conditions in combination with dCas9, followed by dCas9 removal by Cre recombinase as assayed by RT-qPCR and normalized to Actb expression (n = 9 independent clones for gRNAscr, n = 8 independent clones for gRNATnf2). * indicates statistically significant difference of $P < 0.05$, ** of $P < 0.01$, *** of $P < 0.001$, **** of $P < 0.0001$, and ns = not significant (Student's t-test, two-sided, with Holm-Sidak correction if number of tests is greater than 3). Source data are provided as a Source Data file.

Supplementary Figure 10

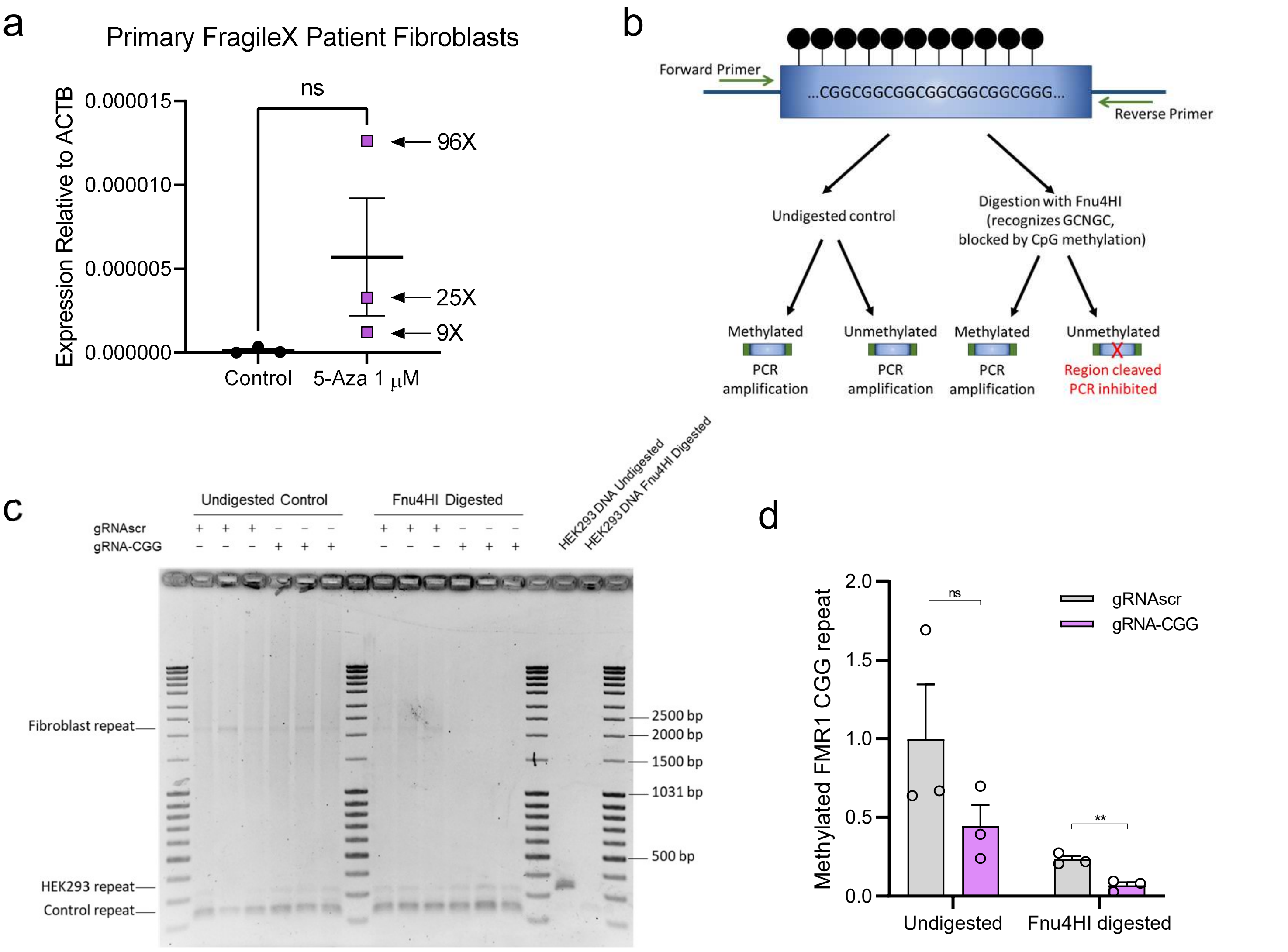

Supplementary Figure 10. Demethylation of *FMR1* promoter. (A) Expression of the *FMR1* gene (mean ± SEM) relative to *GAPDH* expression in primary fibroblasts from Fragile X patient, measured by RT-qPCR after control (water) or 5-aza-2'-deoxycitidine treatment. The difference is not statistically significant (n = 3 biologically independent experiments, P = 0.1, Mann-Whitney test, two-sided). (B) Schematic of the experimental workflow used to determine methylation status of the *FMR1* CGG repeat region. DNA from Fragile X patient fibroblasts is first either subjected to digestion with the methylation sensitive restriction enzyme Fnu4HI or a control (no enzyme) reaction, followed by DNA cleanup and amplification by primers that sit immediately 5' and 3' of the CGG repeat region. A reduction in methylation can be observed by increased DNA digestion by Fnu4HI and failure to amplify by PCR as visualized by agarose gel electrophoresis. (C) Agarose gel electrophoresis results after PCR for the workflow in (B). Lanes 1, 8, 15, and 18 contain MassRuler^{TM} DNA Ladder Mix (Thermo Fisher) and select band sizes are labeled to the right of the gel. Samples are DNA extracted from Fragile X patient fibroblasts expressing dCas9 and either gRNA-CGG or gRNAscr as indicated above the gel. DNA is either undigested control (left 6 samples) or Fnu4HI digested (right 6 samples). Undigested HEK293 control DNA with a shorter repeat region and Fnu4HI digested HEK293 control DNA after PCR are in lanes 16 and 17, respectively. Amplicon size in Fragile X patient fibroblasts is labeled to the left of the gel between 2000 and 2500 bp. Amplicon size in control HEK293 cells is between 300 and 400 bp and is labeled to the left of the gel. Spike-in control DNA to ensure successful PCR is also labeled to the left. (D) Quantification of results in (C) achieved by normalization of intensity of genomic fibroblast amplicon to that of control amplicon using ImageJ software; data is expressed as intensity relative to the undigested dCas9:gRNAscr condition (mean ± SEM). ** indicates statistical significance at $P < 0.01$ and ns indicates no statistically significant difference by Student's t-test,two-sided (n = 3 biological replicates). Source data are provided as a Source Data file.

Supplementary Figure 11

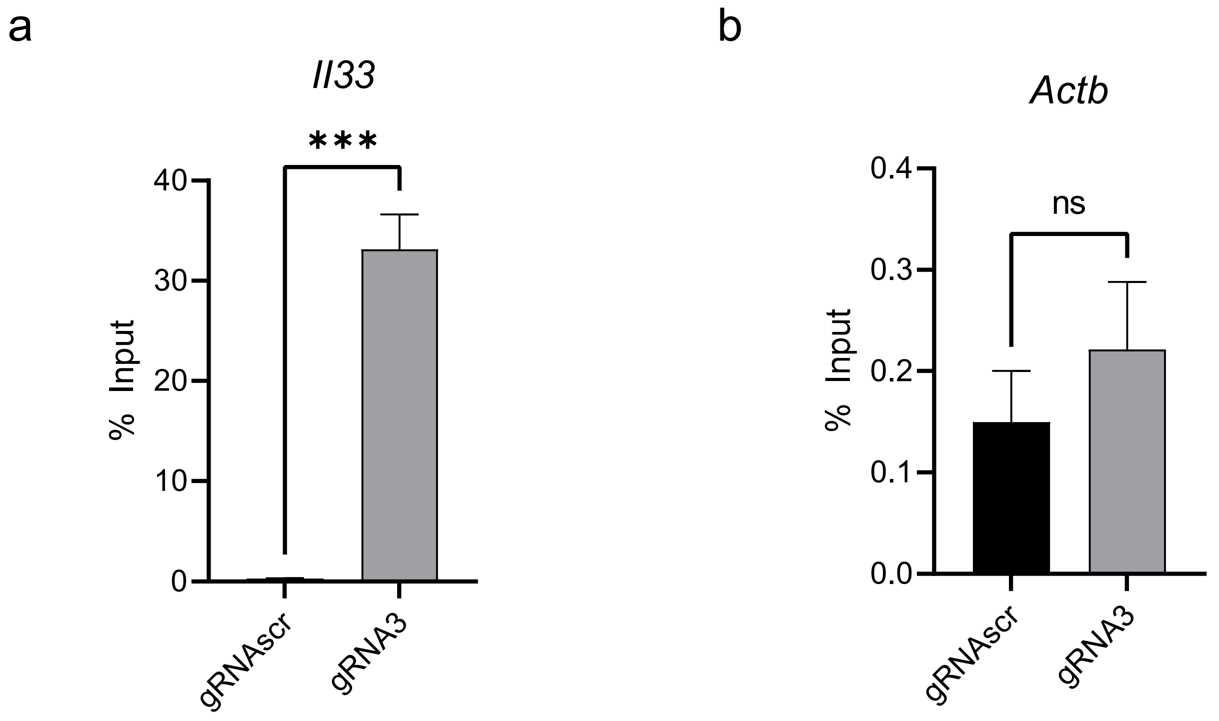

Supplementary Figure 11. Validation of ChIP with anti-FLAG antibody prior to ChIP-seq. (A) Pulldown of *Il33-002* locus measured by qPCR of an amplicon near the transcription start site following ChIP with anti-FLAG antibody in n = 3 independent NIH-3T3 cell lines expressing FLAG-tagged dCas9 and either gRNAscr or gRNA3. Data is expressed as percent input (mean ± SEM) (B). Same as (A) except primers were used for the *Actb* locus, which should not be bound by FLAG-tagged dCas9 in either of the two treatment groups. *** indicates $P < 0.001$ and ns indicates no statistical significance using the two-sided Student's t-test. Source data are provided as a Source Data file.

Supplementary Table 1. Target sequences of all gRNAs used in the study.

Il33 gRNA 1	GAGCCGGTGTTTTCTTGAGC
Il33 gRNA 2	GGTGTGACATAGCCCCATAG
Il33 gRNA 3	GGACTGTGTTAGCTCTCCAC
Il33 gRNA 4	GCTGTGTTAGCTCTCCACCG
Il33 gRNA 5	GCACTCACCTCAATACAGAC
Il33 gRNA 6	GAGCTGATAGATGCTACTAT
scrambled gRNA	GCACTACCAGAGCTAACTCA
HNF4A gRNA	GGGCGCGTTCACGCTGACCA
SERPINB5 gRNA	GAGGAGTGCCGCCGAGGCG
p16/CDKN2A gRNA (CpG 17)	GCATGGAGCCTTCGGCTGAC
SA-gRNA1 (CDKN2A)	AGCAGCATGGAGCCGGCGGCG
SA-gRNA2 (CDKN2A)	GAGCAGCATGGAGCCGGCGGC
SA-gRNA3 (CDKN2A)	GGAGCAGCATGGAGCCGGCGG
SA-gRNA4 (CDKN2A)	AGCGGGCGGCGGGGAGCAGCA
SP-gRNA1 (CDKN2A)	GCAGCATGGAGCCGGCGGCG
SP-gRNA2 (CDKN2A)	AGCAGCATGGAGCCGGCGGC
SP-gRNA3 (CDKN2A)	GAGCAGCATGGAGCCGGCGG
SP-gRNA4 (CDKN2A)	GCGGGCGGCGGGGAGCAGCA
gRNA-CGG (FMR1)	GGCGGCGGCGGCGGCGGCGG
gRNATnf2	GGAGAAGAAACCGAGACAG
gBlock sequence<br>(20bp poly-N is replaced with gRNA sequence)	TGTACAAAAAAGCAGGCTTTAAAGGAACCAATTCAGTCGACT<br>GGATCCGGTACCAAGGTCGGGCAGGAAGAGGGCCTATTTCC<br>CATGATTCCTTCATATTTGCATATACGATACAAGGCTGTTAGA<br>GAGATAATTAGAATTAATTTGACTGTAAACACAAAGATATTA<br>GTACAAAATACGTGACGTAGAAAGTAATAATTTCTTGGGTA<br>GTTTGCAGTTTTAAAATTATGTTTTAAAATGGACTATCATATG<br>CTTACCGTAACTTGAAAGTATTTCGATTTCTTGGCTTTATATA<br>TCTTGTGGAAAGGACGAAACACCNNNNNNNNNNNNNNNNN<br>NNNNGTTTTAGAGCTAGAAATAGCAAGTTAAAATAAGGCTA<br>GTCCGTTATCAACTTGAAAAAGTGGCACCGAGTCGGTGCTTT<br>TTTTCTAGACCCAGCTTTCTTGTACAAAGTTGGCATTA

Supplementary Table 2. Names and sequences of oligonucleotide primers used in this study.

Application	Primer Name	Primer Sequence
In vitro gRNA synthesis	Il33gRNA1_IVT_F	TAATACGACTCACTATAGAGCCGGTGTTTTCTTGAGC
	Il33gRNA1_IVT_R	TTCTAGCTCTAAAACGCTCAAGAAAACACCGGCT
	Il33gRNA2_IVT_F	TAATACGACTCACTATAGGTGTGACATAGCCCCATAG
	Il33gRNA2_IVT_R	TTCTAGCTCTAAAACCTATGGGGCTATGTCACAC
	Il33gRNA3_IVT_F	TAATACGACTCACTATAGGACTGTGTTAGCTCTCCAC
	Il33gRNA3_IVT_R	TTCTAGCTCTAAAACGTGGAGAGCTAACACAGTC
	Il33gRNA5_IVT_F	TAATACGACTCACTATAGCACTCACCTCAATACAGAC
	Il33gRNA5_IVT_R	TTCTAGCTCTAAAACGTCTGTATTGAGGTGAGTG
	Il33gRNA6_IVT_F	TAATACGACTCACTATAGAGCTGATAGATGCTACTAT
	Il33gRNA6_IVT_R	TTCTAGCTCTAAAACATAGTAGCATCTATCAGCT
	gRNAscrambled_IVT_F	TAATACGACTCACTATAGCACTACCAGAGCTAACTCA
	gRNAscrambled_IVT_R	TTCTAGCTCTAAAACTGAGTTAGCTCTGGTAGTG
	p16_IVT_gRNA1F	TAATACGACTCACTATAGCATGGAGCCTTCGGCTGAC
	p16_IVT_gRNA1R	TTCTAGCTCTAAAACGTCAGCCGAAGGCTCCATGC
gBlock amplification and A-tailing	gBlockgRNA_F	TGTACAAAAAAGCAGGCTTTAAAG
	gBlockgRNA_R	TAATGCCAACTTTGTACAAGAAAG
Cre Recombination – Removal of dCas9	dCas9_recomb_F	ATCGTTTCAGACCCACCTCC
	dCas9_recomb_R	AAGCAGCGTATCCACATAGC
Bisulfite PCR and pyrosequencing	Il33_CpGs_1-4_F	(5' biotin)TTTAATTTATAAGATTGAAAGTAGAAAATA
	Il33_CpGs_1-4_R	ACTCTAAACCTTTAAAAAAACACTC
	Il33_CpGs_5-6_F	(5' biotin)TTTGTAATAAGATTTGATATTTTTTTT
	Il33_CpGs_5-6_R	TATTTTATTTTATTCTTTTATTTCTTTCTT
	Il33_CpGs_7-11_F	(5' biotin)TATTTGTTTTAAAAGTTATATTTAAAAGTT
	Il33_CpGs_7-11_F	ACTATACTTTCCTACAATAAACCCC
	p16_bisPCR_fwd_F	TTTTGATTTTAATTTTTTTGTAAATTT
	p16_bisPCR_fwd_R	TCCCCTTACCTAAAAAAATACC
	p16_bisPCR_rev_F	GGAGGGGTTGGTTGGTTATTA
	p16_bisPCR_rev_R	CTTCTAAAAACTCCCCAAAAAAC
	OFF_TARGET_1_F	(5' biotin)GAAGTTGTTGTTAGTTTAGGAGGT
	OFF_TARGET_1_R	CCCCCTTACAAATAAATTCC
	OFF_TARGET_2_F	(5' biotin)TGTGGTTGAGTAAGTGGTAGATATGTT
	OFF_TARGET_2_R	AATCATCTAATTACCCAAATACACC
	OFF_TARGET_3_F	(5' biotin)GTTTGTTTTTTTTGTGTGGAGAGTT
	OFF_TARGET_3_R	CTACATCATTTACAACCCTAAAACCA
	OFF_TARGET_4_F	(5' biotin)TATTTTTTTTAATTTTTTATTTTTTTAAAT
	OFF_TARGET_4_R	TATATTAATTCCCCAATAATTCTTC
	SERPINB5_bisPCR_F	(5' biotin)TTGTTAAGAGGTTTGAGTAGGAGAG
	SERPINB5_bisPCR_R	CCCACCTTACTTACCTAAAATCACA
	HNF4A_bisPCR_F	TTTTTAAGTGATTGGTTATTTTTTAA
	HNF4A_bisPCR_R	ATATCCCATAACCTCCCAAAACTA
	HNF4A_upstream_bis_F	TTTGGAGTTATAAAATTTAATTTAGGTTG
	HNF4A_upstream_bis_R	AAATAACCAATCACTTAAAAAACCC
	HNF4A_dnstream_bis_F	TAGTTTTGGGAGGTTATGGGATAT

	HNF4A_dnstream_bis_R	ACCCCACCCTCTATAAAATTTTAAA
	TNF_pyro_F	/5biosg/TAGATTGTTATAGAATTTTGGTGGG
	TNF_pyro_R	TTCTATTCTCCCTCCTAACTAATCC
Sequencing primers (pyrosequencing)	Il33_CpGs_1-3	TCCTACTACAAATACTTCTTAAA
	Il33_CpG_5	TCCTCTATAAAACTATATCACAC
	Il33_CpGs_9-11	ACTATACTTTCCTACAATAAACC
	OFF_TARGET_1	AAAACAAACCAAAATAACCAACC
	OFF_TARGET_2	CACCCAATACAAAACTCACACAA
	OFF_TARGET_3	CATCATTTACAACCCTAAAACCAA
	OFF_TARGET_4	TATTAATTCCCCAATAATTC
	SERPINB5_CpGs_1-6	CCCACTACCAACCCAACTCC
	TNFpseqnew1-2	AAAACACCCAAACATCAAAA
	TNFpseqnew3-5	AATAACCCTACACCTCTATC
	TNFpseqnew6-7	AAAACTCTCATTCAACCC
	TNFpseqnew8	AACTTCTACTAACTAACTATACA
	TNFpseqnew9-11	TCTCCCTCCTAACTAATCCCTT
gRNA mutagenesis	SERPINB5_MUT_F	GGCACTCCTCCGGTGTTTCGTCCTT
	SERPINB5_MUT_R	GCCGAGGCGGTTTTAGAGCTAGAA
	gRNATnf2_MUT_F	CCGAGACAGGTTTTAGAGCTAGAAATAGCAAG
	gRNATnf2_MUT_F	TTTCTTCTCCCGGTGTTTCGTCCTTTCC
RT-qPCR (where relevant, h = human, m = murine. h/m = human and murine)	mIl33_001_F	AGAAATCACGGCAGAATC
	mIl33_001_R	GTTGGGATCTTCTTATTTTG
	mIl33_002_F	GCTATTTCCTGTCTGTATTG
	mIl33_002_R	TTCTTTGGTCTTCTGTTG
	h/mGAPDH_F	TGCACCACCAACTGCTTA
	h/mGAPDH_R	GGATGCAGGGATGATGTT
	mACTB_F	GGCTGTATTCCCCTCCATCG
	mACTB_R	CCAGTTGGTAACAATGCCATGT
	allgRNA_F	TGTGGAAAGGACGAAACACC
	allgRNA_R	CGGTGCCACTTTTTCAAGTT
	dCas9_blast_F	GATAAGAACCTGCCCAACGA
	dCas9_blast_R	TTTCTCATTCCCTCGGTCAC
	hmaspin_F	ATAACTGTGACTCCAGGCCC
	hmaspin_R	AGAAGAGGACATTGCCCAGT
	hHNF4A_F	GGCCATGGTCAGCGTGAA
	hHNF4A_R	TTCTGATGGGGACGTGTCGTA
	SL-606_hFMR1_qPCR_F	CAGGGCTGAAGAGAAGATGG
	SL-607_hFMR1_qPCR_R	ACAGGAGGTGGGAATCTGA
	mTNF_qPCR_F	GTAGCCCACGTCGTAGCAAA
	mTNF_qPCR_R	TTGAGATCCATGCCGTTGGC
qPCR	Il33_qChIP_9,10,11_F	CCAAAGTTGTTTAATCTGAGCTACC
	Il33_qChIP_9,10,11_R	GGAAATAGCTGGTCTTGAATGC
	pCpGl_luc_qPCR_F	ACCATTGCCTTCACTGATGC
	pCpGl_luc_qPCR_F	TCCTGTGGTTGGTGTTCAGT
	Actb_gDNA_F	GCCACTCGAGCCATAAAAGG
	Actb_gDNA_R	CAAAAGGAGGGGAGAGGGG

Sanger PCR and sequencing primers	pBABE 3'	ACCCTAACTGACACACATTCC
	TET_mutcheck_F	CTTCTCTGGGGTCACTGCTT
	TET_mutcheck_R	CATCGCAGCCCTCTTCTTTC
	TETmutcheck_seq1	TCGATGGCCCCAGATTTGAT
	TETmutcheck_seq2	ACACCCAAAGAGCGGTTATC

Supplementary Table 3. Primers for *S. aureus*-based strategy for *in vitro* gRNA transcription.

SA_TemplateR	AAAAAATCTCGCCAACAAGTTGACGAGATAAACACGGCATTTTGCCTTGTTTTAGTAGATT CTGTTTCCAGAGTACTAAAAC
SP_TemplateR	AAAAAAGCACCGACTCGGTGCCACTTTTTCAAGTTGATAACGGACTAGCCTTATTTTAACTT GCTATTTCTAGCTCTAAAAC
T7FwdAmp	GGATCCTAATACGACTCACTATAG
T7RevAmp_SA	AAAAAATCTCGCCAACAAGT
T7RevAmp_SP	AAAAAAGCACCGACTCGG
SA1_TemplateF	GGATCCTAATACGACTCACTATAGGCAGCATGGAGCCGGCGGCGGTTTTAGTACTCTGG
SA2_TemplateF	GGATCCTAATACGACTCACTATAGAGCAGCATGGAGCCGGCGGCGTTTTAGTACTCTGG
SA3_TemplateF	GGATCCTAATACGACTCACTATAGGAGCAGCATGGAGCCGGCGGGTTTTAGTACTCTGG
SA4_TemplateF	GGATCCTAATACGACTCACTATAGAGCGGGCGGCGGGGAGCAGCAGTTTTAGTACTCTGG
SP1_TemplateF	GGATCCTAATACGACTCACTATAGCAGCATGGAGCCGGCGGCGGTTTTAGAGCTAGAA
SP2_TemplateF	GGATCCTAATACGACTCACTATAGGCAGCATGGAGCCGGCGGCGTTTTAGAGCTAGAA
SP3_TemplateF	GGATCCTAATACGACTCACTATAGAGCAGCATGGAGCCGGCGGGTTTTAGAGCTAGAA
SP4_TemplateF	GGATCCTAATACGACTCACTATAGCGGGCGGCGGGGAGCAGCAGTTTTAGAGCTAGAA

Supplementary Table 4. Table of all 15 samples on which WGBS was performed, listing the number of CpG-context cytosines covered in each sample and the average read coverage of those cytosines.

	**# CpGs covered**	**Average Coverage of those CpGs**
dCas9Cre_gRNA3_1	40312247	7.1791
dCas9Cre_gRNA3_2	40161948	6.73638
dCas9Cre_gRNA3_3	40126578	6.48471
dCas9Cre_gRNAscr_1	40000845	5.99238
dCas9Cre_gRNAscr_2	40240401	6.7508
dCas9Cre_gRNAscr_3	40248194	6.75135
dCas9TET_gRNA3_1	40182464	6.47765
dCas9TET_gRNA3_2	40718796	9.21089
dCas9TET_gRNA3_3	40309695	6.91347
dCas9TET_gRNAscr_1	40348639	7.0694
dCas9TET_gRNAscr_2	39666464	5.2415
dCas9TET_gRNAscr_3	40026430	6.01691
Untreated_1	40093455	6.09392
Untreated_2	40096678	6.02609
Untreated_3	40123189	6.50818
**Average**	**40177068.2**	**6.630182**

Supplementary Table 5. Summary data for coverage and methylation of CpG 9 in the IL33-002 TSS in dCas9:gRNAscr and dCas9:gRNA3 samples.

	dCas9:gRNAscr			dCas9:gRNA3		
**Replicate**	**1**	**2**	**3**	**1**	**2**	**3**
Methylation	100	90.90909	71.42857	0	13.33333	11.11111
Reads	4	11	7	7	15	9

Supplementary Table 6. Sequences and locations of predicted mismatched off-target sites for Il33 gRNA3.

Off-Target #	Mismatching Target Sequence	Chromosome	Position	Strand	Mismatches
1	CGACaGTGTTAGCaCaCCACAGG	chr17	28576152	+	3
2	CGACTGTGTgAGCTCTgtACTGG	chr14	103608747	+	3
3	CagCTGaGTTAGCTCTCCcCTGG	chr8	30382123	+	4
4	aGACTGTGTTAGtTCcCCAgTGG	chr1	63816122	-	4

Supplementary Table 7. Candidate genes in NIH-3T3 cells for robust induction by 5-aza-2'-deoxycytidine. Expression normalized to *ACTB* and expressed as fold change from water-treated controls. Data is presented as average of three biological replicates (*n* = 3) each with two technical replicates.

Gene	Ct (average of n=3)	Ct (average of n=3)	Normalized to Actb		Fold Change
	5-aza 1 µM	Control	5-aza 1 µM	Control	
Tm9sf4	38.74	37.79	2.78658E-07	2.58E-07	1.1
Tnf	31.34	34.205	4.70645E-05	3.1E-06	15.2
Slc44a1	24.045	22.945	0.007391075	0.007599	1.0
Spata2l	28.915	28.38	0.00025275	0.000176	1.4
Cacna1b	29.735	29.69	0.000143168	7.08E-05	2.0
Fam170a	40.295	40.695	9.48353E-08	3.45E-08	2.8
Nars	20.15	20.16	0.109956134	0.052374	2.1
Atp9a	36.025	39.01	1.82965E-06	1.11E-07	16.5
Mkln1	22.535	22.175	0.021050525	0.012958	1.6
Cenpw	24.515	23.22	0.005336095	0.00628	0.8
Fam20a	27.255	26.325	0.000798732	0.00073	1.1
Msi2	23.55	22.595	0.010416396	0.009685	1.1
Taf3	24.16	23.74	0.006824788	0.00438	1.6
Neb	39.28	37.95	1.91653E-07	2.31E-07	0.8
Foxj1	35.96	35.91	1.91397E-06	9.5E-07	2.0
Brinp3	38.435	38.11	3.4426E-07	2.07E-07	1.7
Mcf2	32.795	32.48	1.7167E-05	1.02E-05	1.7
Il33	35.65	35.14	2.37276E-06	1.62E-06	1.5
Actb	16.965	15.905			
