# Supplementary material for: Unraveling the functional role of DNA demethylation at specific promoters by targeted steric blockage of DNA methyltransferase with CRISPR/dCas9
Source: Nat Commun. 2021 Sep 29;12:5711. doi: 10.1038/s41467-021-25991-9 (PMC8481236; doi:10.1038/s41467-021-25991-9)
Supplement: Supplementary file 3 — Description of Additional Supplementary Files [file 41467_2021_25991_MOESM3_ESM.pdf]

Description of Additional Supplementary Files

File Name: Supplementary Software 1
Description: Custom code used to determine lentiviral integration sites and all example files necessary to run code. All instructions are included in the Virus-Clip User Manual_Modified.docx file. This manual contains original user manual for public software with the changes used in this manuscript appended using the Microsoft Word Track Changes function.

File Name: Supplementary Data 1
Description: List of significantly hypomethylated CpGs (≥5X coverage, q<0.01, methylation difference >25%)in dCas9:gRNA3:Cre compared to dCas9:gRNAscr:Cre, given by their chromosomal location with p-value, q-value, and methylation difference.

File Name: Supplementary Data 2
Description: CCTop-generated list of 13,194 possible alignments to gRNA3 in 100bp regions surrounding all 641 off-targets of dCas9:gRNA3 from Supplementary Table S5, allowing for NAG PAMs. The list is ranked from most similar to least similar to gRNA3; the algorithm combines importance of position in gRNA (closer to PAM is more deleterious to gRNA binding) in addition to number of mismatches. Alignment is displayed as vertical lines (matching bases) or dashes (mismatched bases). Top row demonstrates a representative perfect alignment to the on-target Il33-002 region.

File Name: Supplementary Data 3
Description: Differentially enriched regions from ChIP-seq.

File Name: Supplementary Data 4
Description: Methylation data for 424 of 641 off-target dmCpGs of dCas9:gRNA3 that were ≥5X covered in dCas9:gRNAscr:Cre and untreated cells. Table shows methylation fraction and read coverage for each sample, averages, and p-value and FDR-adjusted p-values for one-sided t-test.
