# Supplementary material for: Unraveling the functional role of DNA demethylation at specific promoters by targeted steric blockage of DNA methyltransferase with CRISPR/dCas9
Source: Nat Commun. 2021 Sep 29;12:5711. doi: 10.1038/s41467-021-25991-9 (PMC8481236; doi:10.1038/s41467-021-25991-9)
Supplement: Supplementary file 8 — Supplementary Software 1 [file 41467_2021_25991_MOESM8_ESM.zip › Virus-Clip User Manual_Modified.docx]

**Please note: this is a modified manual to recreate the files generated in Sapozhnikov & Szyf. All changes from original Virus-Clip instructions are tracked with “Track Changes”. The virus_clip.pl script is the same as the original with the following modifications: (1) all instances of “hg19” replaced with “mm10” ; (2) all instances of “human” replaced with “mouse”.**

**Instructions here are for Unix/Mac. Tested on Linux version 3.10.0-1127.19.1.el7.x86_64.**

**Virus-Clip: a fast and memory-efficient viral integration site detection tool at single-base resolution**

**User Manual**

Section I. Preliminary preparation

Section II. Suggested working procedures

Section III. Output file format

Authors: Daniel WH Ho & Irene OL Ng

Department of Pathology and State Key Laboratory for Liver Research, The University of Hong Kong

Contact: dwhho@hku.hk

Last update: 20 January 2015

**Section I. Preliminary preparation**

**a) Downloading the human and virus reference genome**

**File Requirements:**

**Sequencing reads in FASTQ or FASTQ.GZ format (sample dataset provided as R1.fastq.gz and R2.fastq.gz).**

**Mouse (mm10) genome in FASTA format, bisulfite converted (not provided, see below how to produce).**

**Lentiviral sequences between LTRs that integrate in the genome (file provided for each treatment type). These are further *in silico* bisulfite-converted for alignment (both strands) because data format is whole-genome bisulfite sequencing.**

**(b) Program requirements:**

**Install the following programs. Make sure there is a working version of perl. We recommend using anaconda. By using conda, they will all be added to PATH. Otherwise, must be manually added to PATH.**

**Bismark: conda install -c bismark**

**Trim Galore: conda install -c trim-galore**

**Bowtie2: conda install -c bioconda bowtie2**

**Samtools: conda install -c bioconda samtools**

**Blast: conda install -c bioconda blast**

**ANNOVAR: (http://www.openbioinformatics.org/annovar/)**

User should choose the latest version of ANNOVAR (currently 2015Jan06). Following the instructions from the provider to install (usually just download and tar -xzvf to extract). Go the extracted annovar/ folder and follow the instructions to install the mm10 database under the directory annovar/mousedb. This should be accomplished with the command perl annotate_variation.pl -downdb -buildver mm10 refGene mousedb/.**)**.

**c) Prepare bisulfite-converted mm10 genome FASTA and make BLAST database.**

1. Download the mm10 FASTA format genome. This can be found in NCBI: <https://www.ncbi.nlm.nih.gov/assembly/GCF_000001635.20/>

2) Make sure it is in a folder called mm10.

3) Run bismark_genome_preparation with the command bismark_genome_preparation mm10.

4) Prepare a combined bisulfite converted mm10 sequence with the following command, with the following steps.

Navigate to the Bisulfite_Genome folder within the mm10 folder. Run the command:

cat CT_conversion/genome_mfa.CT_conversion.fa GA_conversion/genome_mfa.GA_conversion.fa > mm10_bis_CT_and_GA.fa

5) Make BLAST database for this bisulfite-converted genome with the following command:

makeblastdb -in mm10_bis_CT_and_GA.fa -dbtype nucl

**d) File location set up**

1. Make one directory where all scripts will be run and all files will be stored. For example, let’s call it virusclip/.

2. Move the virus_clip.pl and virus_clip_dCas9project.sh files to this main folder. Move the genomes/ folder to this folder. Then move the mm10 folder to the genomes folder.

3. Move your downloaded annovar/ folder to this folder.

4. In this folder, create separate subfolders with SAMPLE NAMES (e.g dCas9_Cre_gRNA3_1/, dCas9_Cre_gRNA3_2/, etc.). Move the two paired-end fastq.gz files for each sample into the corresponding sample folder.

4. Move to the main folder, and run the script with the following command:

sh virus_clip_dCas9project.sh [sample name] [name of corresponding lentivirus sequences in genome folder]

e.g. sh virus_clip_dCas9project.sh dCas9_Cre_gRNA3_3 dCas9CregRNA3

**Section III. virus_clip.out file format (tab-delimited text)**

Column 1 Left element of the integration event

Column 2 Chromosome for the left element

Column 3 Breakpoint position for the left element

Column 4 Sequence for the left element

Column 5 Right element of the integration event

Column 6 Chromosome for the right element

Column 7 Breakpoint position for the right element

Column 8 Sequence for the right element

Column 9 Supporting soft-clipped read count

Column 10 Affected human gene region

Column 11 Affected human gene
