# Supplementary material for: Unraveling the functional role of DNA demethylation at specific promoters by targeted steric blockage of DNA methyltransferase with CRISPR/dCas9
Source: Nat Commun. 2021 Sep 29;12:5711. doi: 10.1038/s41467-021-25991-9 (PMC8481236; doi:10.1038/s41467-021-25991-9)
Supplement: Supplementary file 9 — Reporting Summary [file 41467_2021_25991_MOESM9_ESM.pdf]

nature portfolio

Corresponding author(s): Moshe Szyf

Last updated by author(s): Jul 22, 2021

Reporting Summary

Nature Portfolio wishes to improve the reproducibility of the work that we publish. This form provides structure for consistency and transparency in reporting. For further information on Nature Portfolio policies, see our Editorial Policies and the Editorial Policy Checklist.

Statistics

For all statistical analyses, confirm that the following items are present in the figure legend, table legend, main text, or Methods section.

n/a	Confirmed	
☐	☒	The exact sample size (*n*) for each experimental group/condition, given as a discrete number and unit of measurement
☐	☒	A statement on whether measurements were taken from distinct samples or whether the same sample was measured repeatedly
☐	☒	The statistical test(s) used AND whether they are one- or two-sided<br>*Only common tests should be described solely by name; describe more complex techniques in the Methods section.*
☒	☐	A description of all covariates tested
☐	☒	A description of any assumptions or corrections, such as tests of normality and adjustment for multiple comparisons
☐	☒	A full description of the statistical parameters including central tendency (e.g. means) or other basic estimates (e.g. regression coefficient) AND variation (e.g. standard deviation) or associated estimates of uncertainty (e.g. confidence intervals)
☐	☒	For null hypothesis testing, the test statistic (e.g. *F*, *t*, *r*) with confidence intervals, effect sizes, degrees of freedom and *P* value noted<br>*Give P values as exact values whenever suitable.*
☒	☐	For Bayesian analysis, information on the choice of priors and Markov chain Monte Carlo settings
☒	☐	For hierarchical and complex designs, identification of the appropriate level for tests and full reporting of outcomes
☐	☒	Estimates of effect sizes (e.g. Cohen's *d*, Pearson's *r*), indicating how they were calculated

Our web collection on statistics for biologists contains articles on many of the points above.

Software and code

Policy information about availability of computer code

Data collection	Pyrosequencing data was collected with Pyromark Q24 software (Qiagen). Western blot images were acquired on the GE Amersham Imager 600. Agarose gel images were acquired with Molecular Imager® Gel Doc™ XR+ (Bio-Rad). qPCR data was collected on the the LightCycler ® 480 Instrument II.
Data analysis	WGBS base calling was performed with RTA v3.4.4 . Program bcl2fastq2 v2.20 was then used to demultiplex samples and generate FastQ reads. Paired-end FastQ files were trimmed for adapters and quality scores using TrimGalore v0.6.4_dev [130] under default settings. Alignments to the mouse mm10 genome, deduplication, and methylation calling were performed using Bismark v0.22.3 [133] under default settings. All statistical analyses were performed with the R package methylKit v1.14.2 [134]. For off-target analyses for dCas9:gRNA3:Cre, significantly differentially methylated (q<0.01, methylation difference >25%) CpGs were determined by comparison to dCas9:gRNAscr:Cre with the calculateDiffMeth function after filtering for CpGs that were covered at least 5X in all samples. Off-target site manhattan plot generated with R package qqman [135].<br><br>Program bcl2fastq2 v2.20 was then used to demultiplex samples and generate fastq reads. Paired-end FastQ files were trimmed for adapters and quality scores using TrimGalore v0.6.4_dev [130] under default settings. Alignments to the mm10 genome were performed using bowtie2 v2.3.4.1 [131] under default settings and peak calling for each sample was performed with the macs2 v2.2.7.1 [132] callpeak function (--g mm --nomodel --extsize 204 --SPMR) after first running the predictd script and establishing --extsize 204 according to the macs2 manual. Alignments were passed to the DiffBind R package to identify significantly differentially enriched regions under default parameters.<br>Viral integration sites were defined by following a pipeline developed by Ho et al. [137] with several key modifications. First, quality trimmed WGBS reads (from above) were aligned with bowtie2 v2.3.4.1 (--very-sensitive-local option) to custom FASTA files containing in silico bisulfite converted sequences (CG to YG, C to T) of forward and reverse strands of the integration-capable lentiviral elements (between two LTRs) from all treatments for that particular cell line: dCas9 plasmids, gRNA3 or gRNAscr plasmids, and Cre plasmids. Notably, the sequence from the lentiviral dCas9 plasmid sequence was in silico recombined (deletion between loxP sites, leaving one loxP site) to mimic Cre action in the cells. Then samtools v1.3.1 [138] was invoked to extract all aligned soft-clipped reads; these are reads that were clipped in order to align to the lentiviral sequences and therefore the clipped portion represents possible read-through into mouse genome (no difference from Ho et al.).

We then ran a modified variant of the script published by Ho et al. (to allow for alignment to mouse bisulfite converted genomic sequences generated by Bismark rather than human unconverted genomic sequences) that used BLAST [139] to identify boundaries between viral and mouse sequences. All overlaps with DMRs were performed with BEDTools intersect v2.29.2 [140].

BISMA (http://services.ibc.uni-stuttgart.de/BDPC/BISMA/) was used to generate Figures 2 and S2C.

Integrated Genome Viewer (https://software.broadinstitute.org/software/igv/download) was used for genome browser displays.

All data involving simple statistical tests not described in WGBS and ChIP-seq methodology (e.g. T-test, Mann-Whitney test, Pearson's r, Holm-Sidak correction for multiple testing) were calculated and graphed with Graphpad Prism 8 software.

Roche Lightcycler 480 software was used for qPCR quantification.

Image J v1.43u was used for quantification of western blots and agarose gels.

For manuscripts utilizing custom algorithms or software that are central to the research but not yet described in published literature, software must be made available to editors and reviewers. We strongly encourage code deposition in a community repository (e.g. GitHub). See the Nature Portfolio guidelines for submitting code & software for further information.

Data

Policy information about availability of data

All manuscripts must include a data availability statement. This statement should provide the following information, where applicable:

- Accession codes, unique identifiers, or web links for publicly available datasets
- A description of any restrictions on data availability
- For clinical datasets or third party data, please ensure that the statement adheres to our policy

The whole genome bisulfite sequencing (WGBS) data generated in this study have been deposited in the Gene Expression Omnibus (GEO) database under accession code GSE162138 (https://www.ncbi.nlm.nih.gov/geo/query/acc.cgi?acc=GSE162138). The chromatin immunoprecipitation sequencing (ChIP-seq) data generated in this study have been deposited in the GEO database under accession code GSE174275 (https://www.ncbi.nlm.nih.gov/geo/query/acc.cgi?acc=GSE174275). Source data are provided with this paper. Mouse mm10 genome is available at https://www.ncbi.nlm.nih.gov/assembly/GCF_000001635.20/.

Field-specific reporting

Please select the one below that is the best fit for your research. If you are not sure, read the appropriate sections before making your selection.

☒ Life sciences ☐ Behavioural & social sciences ☐ Ecological, evolutionary & environmental sciences

For a reference copy of the document with all sections, see nature.com/documents/nr-reporting-summary-flat.pdf

Life sciences study design

All studies must disclose on these points even when the disclosure is negative.

Sample size	Sample sizes for all major data were at least 3, which is typical for experiments with cultured cells. Sample sizes were established as such because triplicate biological experiments are the minimum commonly accepted sample size. Larger sample size determinations used in animal or clinical studies do not apply to in vitro experiments.
Data exclusions	Data that was excluded from analyses was limited to low-quality sequencing as indicated by Illumina quality scores (default trim-galore settings for quality trimming) or pyrosequencing quality report.
Replication	All data is in this study has been replicated in at least three independent biological samples. In some cases (i.e. demethylation of Il33 by gRNA3), the findings have been repeated >20 times. All replication attempts have been successful.
Randomization	Randomization in this study was done when assigning samples to wells in 96-well plate for next-generation sequencing. Allocation of samples to experimental groups is not relevant to this data as this is neither clinical nor animal work. In cell culture work, even different samples originate from the same clonal parental cell line. Cell culture plates were split evenly for all experiments and resulting plates were treated at random.
Blinding	There was no blinding in this study as it is in vitro work with data obtained by instruments not observers and thus does not require blinding. The experimental work was also performed by one person so blinding would not be possible.

Reporting for specific materials, systems and methods

We require information from authors about some types of materials, experimental systems and methods used in many studies. Here, indicate whether each material, system or method listed is relevant to your study. If you are not sure if a list item applies to your research, read the appropriate section before selecting a response.

Materials & experimental systems

n/a	Involved in the study	
☐	☒	Antibodies
☐	☒	Eukaryotic cell lines
☒	☐	Palaeontology and archaeology
☒	☐	Animals and other organisms
☒	☐	Human research participants
☒	☐	Clinical data
☒	☐	Dual use research of concern

Methods

n/a	Involved in the study	
☐	☒	ChIP-seq
☒	☐	Flow cytometry
☒	☐	MRI-based neuroimaging

Antibodies

Antibodies used

1) mouse Anti-CRISPR-Cas9 primary antibody [7A9-3A3] (abcam, ab191468); 2) monoclonal Anti-β-Actin primary antibody produced in mouse (Sigma, A2228); 3) goat Anti-Mouse IgG H&L (HRP) secondary antibody (abcam, ab205719); 4) Monoclonal ANTI-FLAG® M2 antibody (Sigma, F1804); 5) Rabbit IgG, polyclonal - Isotype Control (ChIP Grade) (abcam, ab171870).

Validation

1) For western blot, untreated cells served as negative control for Anti-CRISPR-Cas9 antibody, whereas recombinant dCas9 protein spiked into untreated cell lysate was used as positive control. 2) No validations were performed in this study for monoclonal anti-beta-actin antibody. This is a highly-referenced antibody and a knockout validated antibody (DOI: 10.1016/j.plasmid.2018.08.005). 4) The monoclonal ANTI-FLAG M2 antibody is a gold standard antibody in the field and has been validated hundreds of times. It is targeted towards the synthetic FLAG peptide sequence. The ChIP-seq data published within this manuscript supports this, as the most enriched locus was the Il33 locus targeted by FLAG-tagged dCas9. We also performed ChIP qPCR in the same cells to validate this antibody and IP protocol. It is included in the manuscript as Supplementary Figure S11.

Eukaryotic cell lines

Policy information about cell lines

Cell line source(s)

NIH-3T3 (ATCC), HEK293T (ATCC), Fragile X syndrome fibroblasts (GM05848, Coriell Institute), control fibroblasts (GM00357, Coriell Institute).

Authentication

Cell lines were not authenticated as they were purchased directly from suppliers.

Mycoplasma contamination

Cell lines were not tested for mycoplasma contamination.

Commonly misidentified lines
(See ICLAC register)

No commonly misidentified cell lines were used in this study.

ChIP-seq

Data deposition

☒ Confirm that both raw and final processed data have been deposited in a public database such as GEO.

☒ Confirm that you have deposited or provided access to graph files (e.g. BED files) for the called peaks.

Data access links
May remain private before publication.

https://www.ncbi.nlm.nih.gov/geo/query/acc.cgi?acc=GSE174275

Files in database submission

GSM5290737 gRNA3_1_ANTI_FLAG
GSM5290738 gRNA3_2_ANTI_FLAG
GSM5290739 gRNA3_3_ANTI_FLAG
GSM5290740 gRNA3_1_INPUT
GSM5290741 gRNA3_2_INPUT
GSM5290742 gRNA3_3_INPUT
GSM5290743 gRNAscr_1_ANTI_FLAG
GSM5290744 gRNAscr_2_ANTI_FLAG
GSM5290745 gRNAscr_3_ANTI_FLAG
GSM5290746 gRNAscr_1_INPUT
GSM5290747 gRNAscr_2_INPUT
GSM5290748 gRNAscr_3_INPUT

Genome browser session
(e.g. UCSC)

IGV

Methodology

Replicates

There are three biological replicates for each of two treatments, including three biological replicates of INPUT for normalization.

Sequencing depth	Average total number of reads for each sample was 32 million (minimum 20 million, maximum 42 million). The reads were paired end 100bp, with average duplicate fraction of 25% and average quality of 35-36.
Antibodies	Monoclonal ANTI-FLAG® M2 antibody produced in mouse (Sigma Aldrich catalog no. F1804).
Peak calling parameters	Peak calling for each sample was performed with the macs2 v2.2.7.1 [132] callpeak function (--g mm --nomodel --extsize 204 --SPMR) after first running the predictd script and establishing --extsize 204 according to the macs2 manual
Data quality	DiffBind R package was used to identify significantly differentially enriched regions under default parameters. There were a total of 151 differentially enriched regions in dCas9:gRNA3 and 44 in dCas9:gRNAscr (FDR<0.05). There was no fold change cutoff as this is not a statistical method, however >10% of FDR<0.05 had a fold change >5.
Software	The libraries were normalized and pooled and then denatured in 0.05N NaOH and neutralized using HT1 buffer. The pool was loaded at 200pM on a Illumina NovaSeq S4 lane using Xp protocol as per the manufacturer's recommendations. The run was performed for 2x100 cycles (paired-end mode). A phiX library was used as a control and mixed with libraries at 1% level. Base calling was performed with RTA v3.4.4 . Program bcl2fastq2 v2.20 was then used to demultiplex samples and generate fastq reads. Paired-end FastQ files were trimmed for adapters and quality scores using TrimGalore v0.6.4_dev [130] under default settings. Alignments to the mm10 genome were performed using bowtie2 v2.3.4.1 [131] under default settings and peak calling for each sample was performed with the macs2 v2.2.7.1 [132] callpeak function (--g mm --nomodel --extsize 204 --SPMR) after first running the predictd script and establishing --extsize 204 according to the macs2 manual. Alignments were passed to the DiffBind R package to identify significantly differentially enriched regions under default parameters. No custom code was used.
